# Effectiveness of Strabismus Surgery in Intermittent Exotropia and Factors Influencing Outcome

**DOI:** 10.3390/jcm13041031

**Published:** 2024-02-11

**Authors:** Svenja Kopmann, Ulrike Grenzebach, Oliver Ehrt, Julia Biermann

**Affiliations:** 1 Department of Ophthalmology, University of Muenster Medical Center, 48149 Muenster, Germany or svenja.kopmann@web.de (S.K.);; 2 Department of Ophthalmology, LMU University Hospital, Ludwig-Maximilians Universität Muenchen, 80539 Muenchen, Germany; oliver.ehrt@med.uni-muenchen.de; 3 Department of Ophthalmology, Klinikum Bielefeld Gem. GmbH, 33604 Bielefeld, Germany

**Keywords:** intermittent exotropia, IXT, strabismus, operation, outcome, surgery, recession, resection, reoperation

## Abstract

Intermittent exotropia (IXT) is known to relapse after surgery. No factors to predict or prevent recurrence are known with certainty. This study investigated surgical outcome, potential influencing factors, and reoperation rate in patients with IXT. Medical records of 537 patients who underwent surgery for IXT from 2000 to 2022 with preoperative angles of exodeviation of 6 to 50 prism diopters (PD) were retrospectively studied. Multivariate regression analyses of factors influencing surgical outcome on postoperative day 1 (POD1) and reoperation rate were performed. A Kaplan–Meier analysis was performed to illustrate the reoperation rate. After the first surgery, 83.8% of patients had a successful surgical outcome on POD1 (esodeviation ≤ 5 PD or exodeviation ≤ 10 PD). Logistic regression analysis revealed that small preoperative angles of exodeviation increased the probability for surgical success. Follow-up data at different times (4 days–20 years) after surgery were available for 176 patients: 40 patients were still in the range of surgical success, 133 patients had exotropia > 10 PD. Of the follow-up patients, 65 (12.1%) underwent reoperation. A total of 8.5% had their reoperation within one year after the first surgery, 52.9% within five years. Cox regression analysis revealed that large preoperative angles of exodeviation, far/near incomitance and alphabet pattern strabismus increased the risk of reoperation. Most patients achieved surgical success on POD1, yet the squint angles often increased after surgery, resulting in reoperation in some patients. Prospective studies are needed for a better assessment of pre-, peri- and postoperative factors for surgical success in IXT.

## 1. Introduction

Intermittent exotropia (IXT) is the most common form of divergent strabismus [1,2,3]. It is characterized by a temporary exotropia with panoramic viewing but without diplopia because the patient suppresses overlapping visual fields. Depending on various factors such as ability to concentrate, fatigue, illness, or illumination, the ability to fuse can vary a lot. Under binocular demands or on command, orthotropia can be achieved and patients often have full stereopsis. Usually, patients have no exceptional refractive error and a deviation too large for prism correction. Thus, IXT is treated by surgery when the patient experiences psycho-social stress, has difficulties in everyday life due to increasing asthenopia or, rarely, stereovision deteriorates. However, there are no clear recommendations or guidelines [4]. Although surgery improves deviation and compensation at first, there are frequent recurrences with the need for further operations [5,6,7,8,9,10,11,12]. 

Many studies have already looked at factors influencing surgical outcome. Some suggest that large preoperative squint angles may have a negative impact on surgical success [5,6,13,14,15,16]. Others say that the age of patients at surgery affects outcome [11,16,17,18,19]. A 2019 review concludes that no factor that strongly influences surgical success has been found [20]. A common problem with many studies is the small number of patients included. Many studies have been done with Asian or American patients and there are few studies from Europe. Inhomogeneity of operation technique and indication further reduce comparability of the available data. There are only few studies evaluating surgery for IXT prospectively [12,21,22,23,24].

Our retrospective study analyses a large homogeneous German population who underwent surgery for IXT. The surgical success on postoperative day 1 (POD1) and factors influencing the outcome will be considered. Furthermore, in a subgroup with long-term follow-up we analyze recurrences. Time to further surgeries and predictive factors will be investigated.

## 2. Patients and Methods

Medical records of patients who underwent surgery for IXT with preoperative angles of exodeviation of 6 to 50 prims diopters (PD, distance) at the Department of Ophthalmology at Muenster University Hospital from January 2000 to June 2022 were retrospectively studied. This study was approved by the ethics committee of the Medical Association of Westfalen-Lippe and the University of Muenster (No.: 2022–418-f-S) and adhered to tenants of the Declaration of Helsinki.

We conducted a search in the electronic patient file system FIDUS (Arztservice Wente GmbH, Darmstadt, Germany) filtering for patients with ICD-codes H50.1 or H50.3 in combination with the surgery-code 5–10. Before FIDUS was implemented at our hospital in 2013, medical records were handwritten, and also included in the study. Patients were included if the medical records clearly indicated that they had IXT and were operated on for it at least once in our hospital. They were excluded if they had any previous surgery for IXT elsewhere, any neurologic disorders, developmental delays, craniofacial syndromes, orbital or ocular abnormalities, or coexistent restrictive or paralytic strabismus. Furthermore, patients were excluded if IXT could not be clearly distinguished from other forms of exotropia such as decompensating exophoria (patients with diplopia), constant (not IXT) exotropia, infantile exotropia, or consecutive exotropia. Lastly, patients with preoperative angles of exodeviation more than 50 PD for distance or near gaze were excluded because these patients needed more than two muscle surgery for surgical success.

### 2.1. Preoperative Examinations

The following preoperative patient characteristics were looked at: gender, age, angle of exodeviation in primary position at distance and near (measured with the alternating prism cover test), binocular vision and stereopsis (examined in various tests including Bagolini striated glasses, Titmus test, Lang I and II test, and TNO-test: the lowest value in arc seconds was recorded), fixation preference, additional alphabet pattern strabismus, best corrected visual acuity at distance (given in logMAR), and cycloplegic spherical equivalent. The type of IXT was further classified into basic type (deviation at distance and near differing by less than 10 PD), convergence insufficiency type (squint angle in near gaze is at least 10 PD larger than in distance gaze), and divergence excess type (squint angle in distance gaze is at least 10 PD larger than in near gaze) using Burians classification [25]. The pseudo-divergence excess type could not be distinguished because of missing data on diagnostic monocular occlusion and the use of +3.00 diopters (D) lenses, which are necessary to distinguish the pseudo-divergence excess type from the true divergence excess type. Compensation was not graded in a standardized way in this retrospective study. Amblyopia was defined as a difference in visual acuity of at least two lines. Anisometropia was defined if there was a difference in spherical equivalent ≥ 1 D.

### 2.2. Surgery

The indication for surgery and dosing were given by two of the authors (UG, JB), who are specialists in the field of strabismology and pediatric ophthalmology. Full but no overcorrection of the squint angle was intended. At the Department of Ophthalmology at Muenster University Hospital, a dosage of 2.625 prism diopters per millimeter (PD/mm) or 1.5 degree per millimeter (°/mm) was targeted. In cases of near/distance incomitance, dosage was targeted at maximal correction of the smaller deviation without causing diplopia after prism adaptation. No diagnostic occlusion or long-lasting prism adaptation to increase the deviation were performed. Usually, patients underwent unilateral RR-method for surgery, rarely other methods were used. In cases of significant alphabet pattern, additional surgery on oblique eye muscles was performed.

### 2.3. Outcome Measures

At POD1, the squint angles in distance and near gaze were measured, as well as binocular vision and stereopsis. The surgical success was assessed by the squint angle in distance gaze. Based on many other studies esodeviation ≤ 5 PD or exodeviation ≤ 10 PD were defined as surgical success [5,6,10,11,14,26,27,28,29,30]. Success rates were only calculated for motor criteria, sensory criteria for surgical success were not defined.

### 2.4. Follow-up

**Figure 1:**
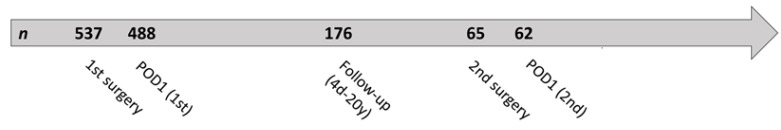
Study design and number of patients considered at different time points. POD1 = postoperative day 1; d = day; y = year.

A total of 176 patients returned to the clinic for follow-up examinations at various times (4 days–20 years) during the course after surgery. Again, the squint angles in distance and near gaze and binocular vision and stereopsis were measured. A total of 65 of these patients underwent multiple surgeries for IXT. Figure 1 shows the study design and the number of patients considered at the various time points.

### 2.5. Statistical Analysis

Statistical analysis was done with IBM SPSS Statistics (IBM statistics for windows, version 28.0., released 2021, Armonk, NY, USA). Paired *t*-tests, *t*-tests, Mann–Whitney U tests, and Pearson’s Chi-squared tests were performed as univariate significance tests. More precisely, Pearson’s Chi-squared tests were calculated for unpaired, categorical variables. For continuous variables, we first checked whether they were normally distributed or not. For unpaired, normally distributed continuous variables, *t*-tests were performed. In contrast, Mann–Whitney U tests were performed for unpaired, non-normally distributed continuous variables. Paired *t*-tests were performed for paired, normally distributed continuous variables. Logistic regression analysis was calculated to find risk factors for poor surgical outcome at POD1. In a first logistic regression model, all selected influencing factors were included, and the backward likelihood ratio method was used to find relevant influencing factors. Then a final logistic regression model was calculated with only the relevant influencing factors included which reduced the number of missing values from 15.6% to 9.7%. Thus, we were able to specify more precisely the odds ratios and 95% confidence intervals (OR and CI). Both logistic regression models are multivariate binary logistic regression analyses, as the outcome variable contains two response categories (surgical success on POD1, yes or no) and several dependent influencing factors are examined. A Kaplan–Meier analysis was performed to illustrate the rate of reoperation. To identify risk factors for reoperation, Cox regression was performed in relation to the initial situation before first surgery. Finally, a log-rank test was performed to examine the differences in reoperation rates in the two groups with successful and unsuccessful surgical outcomes on POD1. For all analyses, a *p*-value <0.05 was statistically significant.

## 3. Results

### 3.1. Patient Characteristics

**Figure 2:**
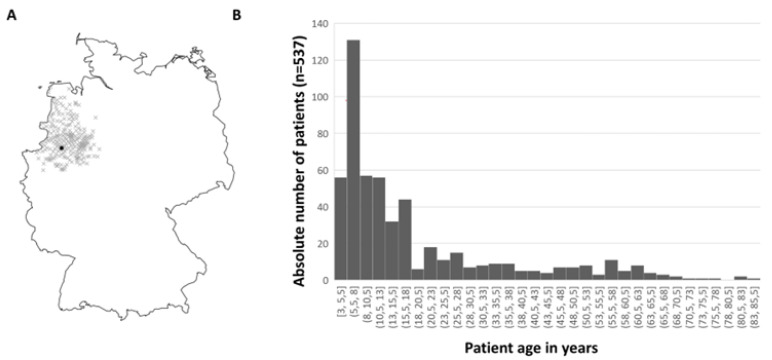
Graphical representation of patient population. (**A**) Residence of patients operated on by ZIP code. On the map of Germany, the patients’ home cities are marked with a cross, the city of Muenster is marked with a black dot. (**B**) Age of patients at first surgery. There is a peak in frequency between the ages of 5.5 and 8 years when children start elementary school.

A total of 537 patients were included in this study. Table 1 shows the patients’ characteristics before the first surgery. A total of 79.8% of the patients had stereopsis with 1200 arc seconds and better. Patients with larger preoperative deviation (≥35 PD) interestingly did not have worse stereopsis than patients with smaller preoperative deviation. Despite the fact that most patients had a strong fixation preference (91.8%), there was a low rate of amblyopia (8.1%). Of the 43 patients with amblyopia, only 12 had anisometropia. A total of 15.2% of the study patients had no refractive error. In contrast, 4.4% of the study patients had a higher refractive error of more than 4.5 D myopia or hyperopia. The remaining 80.4% had a moderate refractive error. Figure 2A shows the home city of all patients, Figure 2B illustrates the age of the patients at first surgery. The entire patient cohort had a mean age of 18.9 years with a standard deviation of 17.3 years. The youngest patient operated on was 3 years old and the oldest patient was 84 years old. Overall, 244 patients (45.4%) were up to ten years old at first surgery. There is a peak in frequency between the ages of 5.5 and 8 years when children start elementary school. At this age, many patients are recommended surgery, firstly so that binocular vision can be used stably as reading activity increases, and secondly to prevent psychosocial stress caused by a visible squint.

### 3.2. Surgical Procedure during First Surgery

526 patients (98.3%) underwent unilateral RR, five patients (0.9%) underwent bilateral lateral rectus recession, and one patient (0.2%) underwent bilateral medial rectus resection. In two of these patients who were not operated on with unilateral RR, a bilateral surgical procedure was chosen because of high far/near incomitance. In the remaining four patients, no rationale for a bilateral surgical procedure was apparent. Because of small preoperative squint angles, three patients (0.6%) underwent unilateral lateral rectus recession. In two patients, no further details on the surgery could be found. The mean value of the operated muscle length in millimeters (mm) was 9 (±2.6 mm, 3 mm–17 mm; *n* = 529). The mean value of the dose–effect ratio was 2.3 PD/mm (±0.9 PD/mm, −0.6 PD/mm −4.7 PD/mm; *n* = 482) or 1.4°/mm, respectively. Thus, there is a slight undercorrection in our collective. The patient with a dose-effect ratio of −0.6 PD/mm was operated on using the unilateral RR method. Unexpectedly, the patient reacted paradoxically to surgery and had larger squint angles in distance and near gaze postoperatively than preoperatively. In the course after the first surgery, the patient was operated on three more times for IXT, always using the unilateral RR method and having preoperative angles of exodeviation at a distance between 25 and 40 PD. In these subsequent operations, a positive dose–effect ratio was always present. It could be confirmed that the first operation was performed correctly. In our collective, there were 20 non-responders in whom divergence surgery had very little effect. In 17 of these patients, the dose–effect ratio was less than 1 PD/mm. In three patients, it was even less than 0.5 PD/mm. In a total of 88 patients (16.4%), divergence surgery was combined with an additional surgery on the oblique eye muscles. This was performed due to V-pattern in 89% of these patients.

### 3.3. Surgical Outcome on POD1 after First Surgery

**Figure 3:**
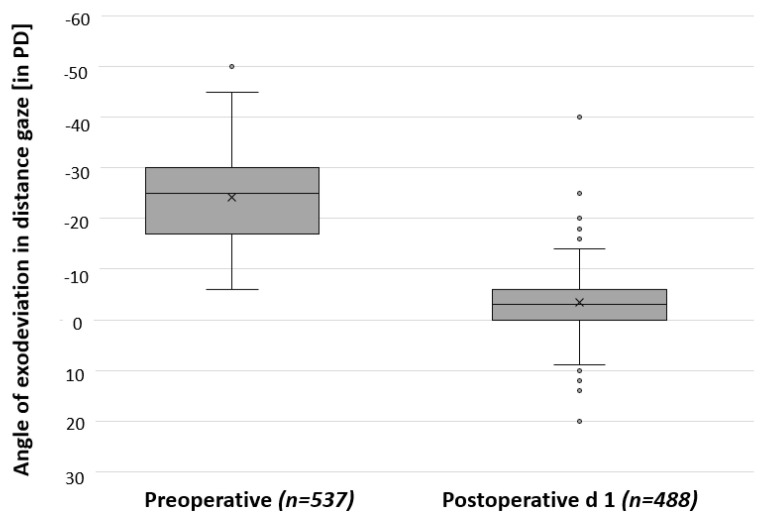
Comparison between squint angles preoperatively and on postoperative day 1 (POD1) after first surgery. Surgery had a significant effect, as indicated by a *p*-value < 0.001 from a paired *t*-test. PD = prism diopters; d = day.

Of 488 patients, the squint angles in distance gaze could be measured on POD1. A total of 409 patients (83.8%) reached surgical success (esodeviation ≤ 5 PD or exodeviation ≤ 10 PD in distance gaze). A total of 44 patients (9.0%) were undercorrected with an exotropia > 10 PD in distance gaze. A total of 35 patients (7.2%) were overcorrected with an esotropia > 5 PD in distance gaze. Figure 3 compares preoperative and postoperative squint angles and illustrates surgical outcome on POD1. Of the 35 patients with overcorrection in distance gaze on POD1, follow-up data were available for 13 patients after a mean period of 1.4 years (±3.4 years). Only two of these patients were still overcorrected in the follow-up examination. No second surgery was required for consecutive esotropia, as these patients were able to compensate well. By contrast, two of the patients who were overcorrected on POD1 required a second surgery for a divergent squint angle over the course of time.

Binocular vision and stereopsis were assessed in 462 patients on POD1: 322 patients (69.7%) had stereopsis of 1200 arc seconds or better. Of the patients who had, preoperatively, stereopsis with worse than 1200 arc seconds, 25.6% had stereopsis of 1200 arc seconds or better on POD1. Thus, these patients improved in stereopsis. However, there were also 16.5% patients who deteriorated.

### 3.4. Risk Factors for Poor Surgical Outcome on POD1 after First Surgery

A logistic regression analysis of factors influencing surgical outcome on POD1 revealed that a small preoperative squint angle increased the probability for successful surgical outcome (*p*-value: <0.001; OR: 1.044 (CI: 1.018–1.071)). An existing far/near incomitance of the squint angles tended to reduce the probability for successful surgical outcome. However, this result was not statistically significant (*p*-value: 0.07; OR: 0.594 (CI: 0.338–1.044)). Age at surgery, preoperative binocular vision and stereopsis, alphabet pattern strabismus, and additional surgery on oblique eye muscles had no influence on the surgical outcome on POD1. Table 2 shows a comparison between the two groups with or without successful surgical outcome on POD1. The results of univariate significance tests align well with the results of the logistic regression analysis. In addition to the information given in the table, there was no statistically significant difference between the two groups with regard to preoperative binocular vision and stereopsis (*p*-value of a Pearson’s chi-squared test: 0.646) and also with regard to refractive errors (*p*-value of a Pearson’s chi-squared test: 0.386).

### 3.5. Surgical Outcome after First Surgery in Follow-up Examinations

**Figure 4:**
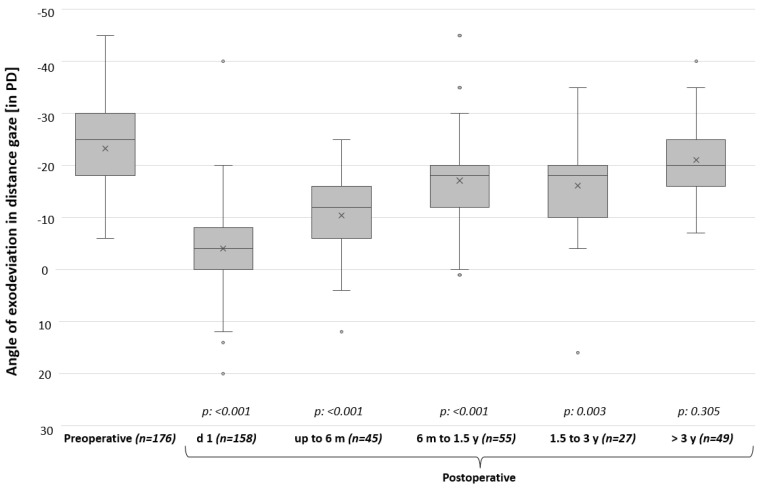
Comparison between squint angles preoperatively, on postoperative day 1 (POD1), and in follow-up examinations at different times after first surgery. Only patients with follow-up data are included in this graph with their last visit. For each patient with follow-up, the preoperative squint angle, the squint angle on POD1 (if available), and one squint angle of latest follow-up examination are listed. Italics show *p*-values from paired *t*-tests comparing preoperative squint angles with squint angles at different time points postoperatively. A significant effect of surgery was observed up to 3 years postoperatively. PD = prism diopters; d = day; m = month; y = year.

Out of the 537 patients who had undergone one surgery, a total of 176 patients came to our institution for follow-up examinations at various times (4 days–20 years) after first surgery: 45 patients (25.6%) came up to six months after first surgery, 55 patients (31.3%) six months to 1.5 years, 27 patients (15.3%) 1.5 to three years, 20 patients (11.4%) three to five years, 17 patients (9.7%) five to ten years and 12 patients (6.8%) came more than ten years after surgery. While 77.2% of these patients with follow-up data had been within the range of surgical success on POD1, only 40 patients (22.7%) fulfilled this definition later on in the follow-up examinations with a mean follow-up time of 2.2 years (±4.1 years). A total of 133 patients (75.6%) had exotropia more than 10 PD in follow-up examinations, the remaining three patients (1.7%) had esotropia more than 5 PD. Figure 4 compares squint angles preoperatively, on POD1 and in follow-up examinations at different times after surgery. It indicates that the squint angles became more divergent again in the course of time in this group of patients who returned for follow-up. In the group of patients who returned after more than three years after surgery, there was no longer a significant effect of surgery. 

Binocular vision and stereopsis were assessed in 156 patients at follow-up examinations: 133 patients (85.3%) had stereopsis of 1200 arc seconds or better. Of the patients who were still within the range of surgical success at the follow-up examination, 81.8% had stereopsis of 1200 arc seconds or better. Of the patients who were not in the range of surgical success in the follow-up examination, 86.2% nevertheless had stereopsis of 1200 arc seconds or better.

### 3.6. Reoperations over the Course of Time after First Surgery

**Figure 5:**
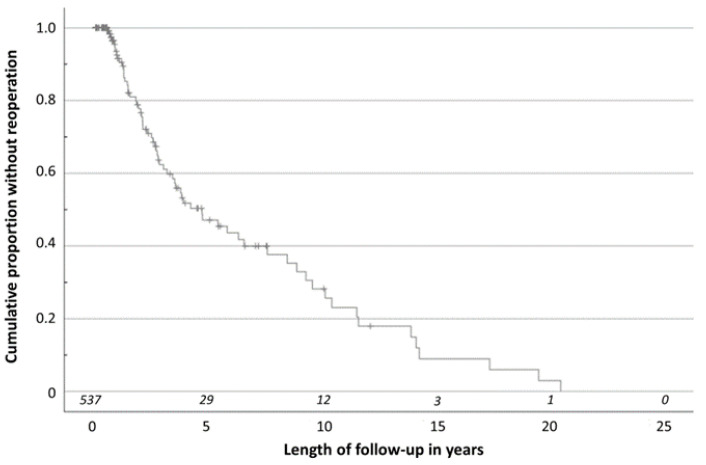
Kaplan–Meier analysis illustrating reoperation rate over time. The number at risk is shown in italics. A total of 8.5% of reoperations were performed within the first year after first surgery, 38.9% within the first three years, and 52.9% within the first five years.

A total of 65 patients (12.1% of all patients included in this study and 36.9% of the patients from whom follow-up data were available) returned for reoperation because of a remaining or recurrent divergent squint angle. Reoperation was performed on the contralateral eye in 50 patients and on the same eye in 15 patients. Of the reoperated patients, 61 underwent a total of two operations for IXT at our hospital, two patients underwent a total of three operations, and two patients underwent a total of four operations. One patient was operated on twice because of IXT and then after the second operation had a consecutive esotropia with diplopia which resulted in a third surgery against the convergent squint angle. Figure 5 shows a Kaplan–Meier analysis which was done to illustrate the reoperation rate over time. Median time to reoperation was 4.7 years (CI: 2.687–6.716 years). According to our Kaplan–Meier analysis, 8.5% of patients required reoperation within one year after first surgery, 38.9% within three years, and 52.9% within five years.

### 3.7. Risk Factors for Reoperations

A Cox regression analysis of factors influencing reoperation rate revealed that a small preoperative squint angle (*p*-value: 0.023; OR: 0.963 (CI: 0.932–0.995)) reduced the risk of reoperation. An existing far/near incomitance of the squint angles (*p*-value: 0.023; OR: 2.527 (CI: 1.134–5.634)) and an alphabet pattern strabismus (*p*-value: 0.026; OR: 1.910 (CI:1.079–3.381)) increased the risk for reoperation. Age at surgery, preoperative binocular vision and stereopsis, additional surgery on oblique eye muscles, and surgical outcome on POD1 had no influence on the reoperation rate. In addition, a log-rank test showed that there was no statistically significant difference looking at reoperation rates in the two groups with and without successful surgical outcome on POD1 (*p*-value: 0.272). Table 3 refers to patients who came to follow-up examinations and compares data before the first surgery of patients with and without reoperation. The results of the univariate significance tests, unlike the Cox regression analysis, showed no statistically significant differences in preoperative squint angles, far/near incomitance, and alphabet pattern strabismus between patients with and without reoperation. In addition, the univariate significance tests also showed that there was no statistically significant difference between the two groups with regard to preoperative binocular vision and stereopsis (*p*-value of a Pearson’s chi-squared test: 0.861) and with regard to refractive errors (*p*-value of a Pearson’s chi-squared test: 0.242). This information is not shown in the table for reasons of space.

### 3.8. Surgical Outcome on Postoperative Day 1 after Second Surgery, POD1(2nd)

Squint angles in distance gaze on POD1(2nd) were available from 62 patients. A total of 48 patients (77.4%) had a successful surgical outcome. Of the patients who did not achieve this, six patients were still undercorrected and eight patients were overcorrected.

## 4. Discussion

This monocentric retrospective cohort study of patients receiving predominantly unilateral RR surgery against IXT performed in Muenster, Germany included a total of 537 patients and is, to our best knowledge, the largest European study on this issue. The main results can be summarized as follows: Almost 84% of patients were in the range of surgical success on POD1 after the first surgery. A smaller preoperative squint angle seemed to significantly increase the likelihood of this. In the 176 patients from whom follow-up data after first surgery were available, it was noticeable that only 40 patients were still in the range of surgical success while 133 patients had exotropia > 10 PD for distance gaze. Of the follow-up patients, 65 underwent reoperation at our institution. A total of 8.5% had their reoperation within one year after the first surgery, 52.9% within five years. Large preoperative squint angles, alphabet pattern strabismus, and far/near incomitance could possibly increase the risk for reoperation during follow-up.

### 4.1. Surgical Success on POD1 and Risk Factors for Poor Surgical Outcome

Other studies also examined surgical success rates and influencing factors. However, studies often have different times for looking at surgical outcomes and partly different criteria defining surgical success. A monocentric retrospective Scandinavian study by Thorisdottir et al. in 2021 that included 190 patients with IXT came to similar conclusions as we did. They found an 80% success rate six weeks postoperatively and only a larger preoperative squint angle as a factor influencing surgical outcome negatively [6]. In a 2016 monocentric retrospective South Chinese study by Yang et al. with a number of 1228 patients included, they found an 80.5% success rate using motor criteria for surgical success after a mean follow-up time of 7.8 months [31]. However, the success rate was also lower in other studies, with Chew et al. including 80 patients and having a success rate of 75% one week postoperatively [5] or Lee et al. including 101 patients and having a success rate of 61.4% one month postoperatively [28]. It is crucial to consider latency period after surgery to draw comparisons. It is well known that squint angles weaken postoperatively after divergence operation. Furthermore, poor vision and improper fixation may impact the results of angle quantification on POD1. While we primarily looked at the surgical success on POD1 (stationary operations, day of hospital discharge), other studies looked at it at a later time. After a longer follow-up period, surgical success was also significantly lower in our study. 

Just like Thorisdottir et al. and us, many other authors reported the relationship between large preoperative squint angles and negative surgical outcomes [5,6,13,14,15,16]. This result is very likely a multifactorial condition and can be a result of measurement inaccuracy, dosing, and other individual factors. On the other hand, Yang et al. did not observe that preoperative squint angles were related to surgical outcome; they found only the loss of stereopsis as a risk factor for poor surgical outcome, which we did not find [31]. Age at surgery as an influencing factor is controversially discussed. There are authors claiming that younger age at surgery is a factor influencing surgical outcome positively [17,18,19], authors who claim that age has no influence [6,32,33], and authors who claim that younger age at surgery is a factor influencing surgical outcome negatively [11,16]. We could not find any influence of age on surgical outcome. Although the result was not statistically significant, our data suggest that patients with existing far/near incomitance of squint angles have worse surgical outcomes. In these patients it is more difficult to plan surgery to avoid over- or undercorrection in distance or near gaze. Bae et al. examined 342 South Korean patients and found that the surgical outcome was better in basic type IXT than in divergence excess type. However, they also found that it was even better in patients with pseudo-divergence excess type, which we could not distinguish in our analysis due to a lack of data [29]. Our results on the investigated risk factors on surgical outcome align well with the results of many other studies that included fewer patients. Prospective studies are urgently needed to confirm this. Defining cut-off levels of PD or squint angles, above which dosing is going to be limited, would be very desirable. Furthermore, surgical success should be valued by other factors than angle size alone in prospective studies, for example, the ability and duration of compensation.

### 4.2. Outcome in Follow-up Examinations: Increasing Squint Angles

Follow-up data were available from 176 patients. In our results, there was a clear tendency that the squint angles became more divergent again in the course of time after surgery. The more time that passed after surgery, the more the patients showed divergent squint angles. Many other studies also reported this postoperative exodrift and its correlation to longer follow-up time [5,6,7,8,9,10,11,12]. This is a huge and unsolved problem in IXT surgery, and it is interesting that this happened independently from angle size directly after surgery. It is therefore tempting to speculate that pre- and intraoperative influencing factors may play a subordinate role.

Some patients underwent reoperation for IXT. However, there were also patients in whom large squint angles were measured in the follow-up examination but did not undergo reoperation. Reasons for not reoperating on these patients were that patients were satisfied with the surgical outcome of the first surgery and did not wish another one. As known from clinical routine, a lot of patients can compensate even large squint angles without problems and have no distress. It is not only the size of the squint angle that is decisive for the indication for surgery, but also other factors such as the frequency of squinting, a threatened loss of binocular vision and stereopsis, and psychosocial reasons must be taken into consideration [4]. Holmes et al. in 2021 stated that successful surgery in particular improves patients’ quality of life, but quality of life was also improved in patients with suboptimal surgical outcomes [34]. The health-related quality of life of patients with IXT seems to be related to stereopsis [35,36,37]. In our collective, there was no major difference in stereopsis between patients who were still in the range of surgical success in follow-up examinations as opposed to patients who were not. This was also shown by other authors [30].

### 4.3. Reoperations

Pineles et al. prospectively reevaluated reoperation rate in a study including 50 patients (of whom 30 patients were reoperated on) with a minimum follow-up time of 10 years. While in our data the median time to reoperation was 4.7 years, in the study of Pineles et al. it was 7.4 years. However, time to reoperation varied widely in both studies. Pineles et al. found postoperative undercorrection and lateral incomitance as risk factors associated with reoperation, while we identified the preoperative squint angle before first surgery, an existing far/near incomitance, and alphabet pattern strabismus as risk factors [24]. It may be important for patients to know at what time reoperation is needed. They may find it more comfortable to know that reoperation is unlikely to be necessary shortly after the first surgery. We found that the probability of reoperation within the first year after surgery is less than 9%.

### 4.4. Limitations

Our study needs to be understood with its limitations. First of all, this is a retrospective cohort study, which in itself is a source of bias and confounding. As a result of the retrospective design, there were no fixed appointments with patients for follow-up examinations and therefore we have a lack of long-term results in many patients. It is possible that mostly patients with large postoperative residual angles were called for follow-up examinations or patients who were dissatisfied with the outcome came on their own decision. This may have caused a bias. It follows that patients came for follow-up examinations at completely different times after surgery. Likewise, some patients may have gone to another hospital to be reoperated on for IXT without our knowledge. This objection can only be hypothetically alleviated as alternative regional strabismus care facilities are rare, as seen in the supply area on the map in Figure 2A. Another potential bias to be deliberated is the subjectivity of measures for stereopsis. More objective measures are available nowadays, such as studying reflexive eye movements [38,39]. These issues should be taken up for prospective studies. In our study, just like in most others, surgical success refers only to motor criteria and no sensory criteria for surgical success were defined. In contrast, some authors have added sensory criteria [9,24,31]. For the sake of completeness, however, then it would be best to also include compensation and patients’ satisfaction after surgery, which was not possible here due to the retrospective design. 

## 5. Conclusions

Despite its limitations, our study examined a large, homogeneous cohort of 537 patients (45.4% ≤ ten years old). The clinical characteristics of our cohort represent real live data, showing that 79.8% of the patients had stereopsis of 1200 arc seconds or better, which was independent from squint angle size, and that amblyopia was a rare finding in IXT. Surgical success on POD1 was, remarkably, not related to reoperation rate. This raises the question of whether or not squint angle amount is itself crucial for decompensation tendency or the duration of squinting periods. In the future, prospective studies with a large number of patients included are needed for a better assessment of the influencing factors before, during, and after surgery.

## Figures and Tables

**Table 1:** Patient characteristics and examination results before first surgery for the 537 patients included in this study.

**Gender ^a^**	
Female	295 (55%)
Male	242 (45%)
**Preoperative angle of deviation in PD ^b^**	
Distance	Exo 24.2 ± 9.6 (Exo 6–Exo 50)
Near (*n = 534*)	Exo 24.8 ± 10.9 (Exo 2–Exo 50)
**Type of exotropia ^a^ (*n = 534*)**	
Basic	437 (81.8%)
Convergence insufficiency	62 (11.6%)
Divergence excess	35 (6.7%)
**Alphabet pattern strabismus ^a^**	
V-pattern	122 (22.7%)
A-pattern	36 (6.7%)
**Preoperative binocular vision and stereopsis ^a^ (*n = 505*)**	
Bagolini test negative	58 (11.5%)
Bagolini test positive	31 (6.1%)
Titmus fly positive	13 (2.6%)
1200–550 arc seconds	130 (25.7%)
549–101 arc seconds	89 (17.6%)
≤ 100 arc seconds	184 (36.4%)
**Fixation preference ^a^ (*n = 426*)**	391 (91.7%)
**Best corrected visual acuity at distance logMAR ^bc^ (*n = 532*)**	0 ± 0.7 (−0.1–0.6 *)
**Amblyopia ^a^ (*n = 531*)**	43 (8.1%)
**Spherical equivalent in D ^bc^ (*n = 479*)**	0.2 ± 2.1 (−8.3–+9.0)
**Anisometropia ≥ 1** **D ^a^ (*n = 479*)**	71 (14.8%)

If no *n* is given in parentheses, the data refer to the total number of patients *n*= 537. ^a^: absolute number (relative frequency); ^b^: mean value ± standard deviation (minimum–maximum); ^c^: mean value of right and left eye; PD = prism diopters; D = diopters; *: due to poor compliance a few children gave subnormal values for visual acuity.

**Table 2:** Comparison of the groups with or without successful surgical outcome on postoperative day 1 (POD1) after first surgery.

	Successful POD1	Not Successful POD1	*p*-Value
	*n = 409*	*n = 79*	
**Age at first surgery in years ^b^**	18.1 ± 16.2 (3–84)	22.9 ± 21.6 (4–83)	0.405 ^e^
**Preoperative angle of deviation in PD ^b^**			
Distance	Exo 23.8 ± 9.5 (Exo 6–Exo 50)	Exo 28.1 ± 8.7 (Exo 6–Exo 50)	<0.001 ^f^
Near	Exo 24.5 ± 10.8 (Exo 3–Exo 50) (*n = 406*)	Exo 29.0 ± 10.4 (Exo 10–Exo 50)	<0.001 ^f^
**Type of exotropia ^a^**	(*n = 406*)	(*n = 79*)	0.035 ^d^
Basic	337 (83%)	56 (70.9%)	
Convergence insufficiency	45 (11.1%)	14 (17.7%)	
Divergence excess	24 (5.9%)	9 (11.4%)	
**Alphabet pattern strabismus ^a^**			0.870 ^d^
V-pattern	91 (22.2%)	18 (22.8%)	
A-pattern	25 (6.1%)	6 (7.6%)	
**Fixation preference ^a^**	304 (92.1%) (*n = 330*)	52 (89.7%) (*n = 58*)	0.603 ^d^
**Best corrected visual acuity at distance logMAR ^bc^**	0 ± 0.7 (−0.1–0.6) (*n = 404*)	0 ± 0.7 (−0.1–0.4) (*n = 78*)	0.074 ^f^
**Amblyopia ^a^**	26 (6.4%) (*n = 404*)	10 (12.8%) (*n = 78*)	0.060 ^d^
**Spherical equivalent in D ^bc^**	0.1 ± 2.0 (−7.3–+8.0) (*n = 366*)	0.4 ± 2.6 (−8.3–+9.0) (*n = 74*)	0.274 ^f^
**Anisometropia ≥ 1 D ^a^**	55 (15%) (*n = 366*)	11 (14.9%) (*n = 74*)	0.882 ^d^
**Operated muscle length in mm ^b^**	8.9 ± 2.6 (4–17) (*n = 403*)	9.6 ± 2.5 (6–16)	0.024 ^f^
**Dose-effect ratio in PD/mm ^b^**	2.3 ± 0.8 (0.4–4.3) (*n = 403*)	2.5 ± 1.2 (−0.6–4.7)	0.237 ^f^
**Type of operation ^a^**	(*n = 407*)		0.211 ^d^
RR	401 (98.5%)	77 (97.5%)	
BLRc	4 (1%)	1 (1.3%)	
BMRs	0 (0%)	1 (1.3%)	
LRc	2 (0.5%)	0 (0%)	
**Additional surgery on oblique eye muscles ^a^**	69 (17%) (*n = 407*)	10 (12.7%)	0.407 ^d^

If no *n* is given in parentheses, the data refer to the *n*-number above. ^a^: absolute number (relative frequency); ^b^: mean value ± standard deviation (minimum–maximum); ^c^: mean value of right and left eye; ^d^: Pearson’s chi-squared test; ^e^: Mann–Whitney U test; ^f^: *t*-test; PD = prism diopters; D = diopters; mm = millimeters; PD/mm = prism diopters per millimeter; RR = unilateral lateral rectus recession with medial rectus resection; BLRc = bilateral lateral rectus recession; BMRs = bilateral medial rectus resection; LRc = unilateral lateral rectus recession.

**Table 3:** Comparison of the groups with or without reoperation in the course after the first surgery.

	No 2nd Surgery	2nd Surgery	*p*-Value
	*n = 111*	*n = 65*	
**Age at first surgery in years ^b^**	14.2 ± 17.5 (4–83)	14.1 ± 13.0 (3–58)	0.111 ^e^
**Preoperative angle of deviation in PD ^b^**			
Distance	Exo 24.0 ± 8.8 (Exo 6–Exo 45)	Exo 22.1 ± 9.0 (Exo 6–Exo 45)	0.180 ^f^
Near	Exo 25.1 ± 9.5 (Exo 5–Exo 50)	Exo 22.1 ± 11.0 (Exo 2–Exo 50)	0.072 ^f^
**Type of exotropia ^a^**			0.801 ^d^
Basic	88 (79.3%)	52 (80%)	
Convergence insufficiency	16 (14.4%)	7 (10.8%)	
Divergence excess	7 (6.3%)	6 (9.2%)	
**Alphabet pattern strabismus ^a^**			0.668 ^d^
V-pattern	26 (23.4%)	19 (29.2%)	
A-pattern	9 (8.1%)	4 (6.2%)	
**Best corrected visual acuity at distance logMAR ^bc^**	0 ± 0.7 (−0.1–0.3) (*n = 110*)	0 ± 0.7 (−0.1–0.6)	0.231 ^f^
**Amblyopia ^a^**	7 (6.4%) (*n = 110*)	2 (3.1%)	0.283 ^d^
**Spherical equivalent in D ^bc^**	0.5 ± 1.9 (−4.3–+6.8) (*n = 99*)	0.7 ± 1.7 (−2.3–+8.0) (*n = 59*)	0.404 ^f^
**Anisometropia ≥ 1 D ^a^**	11 (11.2%) (*n = 99*)	6 (10.2%) (*n = 59*)	0.081 ^d^
**Operated muscle length in mm ^b^**	8.6 ± 2.4 (3–15) (*n = 110*)	8.6 ± 2.2 (4–14)	0.839 ^f^
**Dose-effect ratio in PD/mm ^b^**	2.3 ± 0.9 (0.7–4.7) (*n = 100*)	2.1 ± 0.9 (−0.6–4.1) (*n = 57*)	0.021 ^f^
**Type of operation ^a^**			0.704 ^d^
RR	108 (97.3%)	65 (100%)	
BMRs	1 (0.9%)	0 (0%)	
LRc	2 (1.8%)	0 (0%)	
**Additional surgery on oblique eye muscles ^a^**	30 (27%)	14 (21.6%)	0.474 ^d^
**Angle of deviation POD1 in PD ^b^**			
Distance	Exo 3.6 ± 7.3 (Exo 18–Eso 20) (*n = 101*)	Exo 4.7 ± 7.4 (Exo 40–Eso 6) (*n = 57*)	0.362 ^f^
Near	Exo 6.8 ± 7.4 (Exo 25–Eso 16) (*n = 98*)	Exo 6.9 ± 8.7 (Exo 50–Eso 3) (*n = 58*)	0.943 ^f^
**Surgical outcome POD1 ^a^**	(*n = 101*)	(*n = 57*)	0.188 ^d^
Successful	74 (73.3%)	48 (84.2%)	
Undercorrection	16 (15.8%)	7 (12.3%)	
Overcorrection	11 (10.9%)	2 (3.5%)	

This table refers only to the 176 patients from whom follow-up data are available. Data refer to the first surgery. If no *n* is given in parentheses, the data refer to the *n*-number above. ^a^: absolute number (relative frequency); ^b^: mean value ± standard deviation (minimum–maximum); ^c^: mean value of right and left eye; ^d^: Pearson’s chi-squared test; ^e^: Mann–Whitney U test; ^f^: *t*-test; PD = prism diopters; D = diopters; mm = millimeters; PD/mm = prism diopters per millimeter; RR = unilateral lateral rectus recession with medial rectus resection; BLRc = bilateral lateral rectus recession; BMRs = bilateral medial rectus resection; LRc = unilateral lateral rectus recession; POD1 = postoperative day 1.

## Data Availability

Data are contained within the article.
